# Randomized investigation to evaluate phenylalanine fluctuation after overnight fasting in PKU patients treated with prolonged-release versus standard amino acid protein substitute

**DOI:** 10.1186/s13023-026-04264-y

**Published:** 2026-04-02

**Authors:** Anne Daly, Fatma Ilgaz, Sharon Evans, Catherine Ashmore, Alex Pinto, Anita MacDonald

**Affiliations:** 1 Birmingham Children’s Hospital, Birmingham, B4 6NH UK; 2 Department of Nutrition and Dietetics, Faculty of Health Sciences, Hacettepe University, Ankara, 06100 Turkey

**Keywords:** Phenylketonuria, PKU, Prolonged-release protein substitute, Blood phenylalanine, Phenylalanine fluctuation

## Abstract

**Background:**

Effective management of phenylketonuria (PKU) requires consistent blood phenylalanine (Phe) control. However, patients, particularly those with classical PKU, often experience diurnal fluctuations, particularly with early morning Phe levels after an overnight fast. These fluctuations have been linked to neurocognitive and psychological impairments. This 4-week, randomized, controlled crossover study evaluated the efficacy of a prolonged-release amino acid (PR-AA) compared to a conventional free amino acid-based protein substitute (AA) in reducing early morning blood Phe levels in patients with classical PKU.

**Methods:**

Participants on a low-Phe diet supplemented with protein substitutes were randomly assigned to one of two treatment arms: Arm A, participants received the PR-AA as a single evening dose for 7 days, followed by a 2-week washout period, then crossed over to receive their usual AA protein substitute for all daily doses for another 7 days. Arm B, the order was reversed. All other daily protein substitute doses remained unchanged, using the patients’ usual Phe-free L-AA protein substitute throughout the study. Blood Phe and tyrosine (Tyr) levels, Phe/Tyr ratio, branched-chain amino acids (BCAAs), urinary nitrogen and creatinine excretion, tolerability, and patient acceptability were assessed.

**Results:**

From 16 eligible patients, 13 children with classical PKU (*n=* 8 females, 62%) with a mean age of 11.8 ± 3.2 years completed the study. Administration of the PR-AA significantly reduced early morning Phe levels compared to AA (mean difference from baseline to the end of treatment with PR-AA: 63.6 ± 28.93 µmol/L; −17.8%; *p =* 0.0484; with AA: 95.5 ± 28.9 µmol/L; +27.6%; *p =* 0.0063). Subgroup analyses indicated greater benefit in patients < 12 years and in those with higher baseline Phe level (>360 μmol/L). PR-AA also significantly increased blood Tyr levels, and improved the Phe/Tyr ratio. No significant differences were observed for other parameters. PR-AA was well tolerated, with high adherence.

**Conclusions:**

PR-AA demonstrated improved metabolic control, particularly in reducing early morning Phe levels. PR-AA may be a useful alternative protein substitute for patients with classical PKU, particularly those with poor metabolic control. Further long-term studies are warranted to assess sustained efficacy, adherence, and neurocognitive outcomes.

**Supplementary information:**

The online version contains supplementary material available at 10.1186/s13023-026-04264-y.

## Background

Phenylketonuria (PKU; OMIM 261,600), a rare inherited metabolic disorder with an average global prevalence of 1 in 23,930 live births [1], is caused by mutations in the gene encoding the hepatic phenylalanine hydroxylase (PAH) enzyme [2, 3]. PAH deficiency disrupts the conversion of phenylalanine (Phe) into tyrosine (Tyr), resulting in high blood and cerebral Phe concentrations, with concomitant Tyr depletion [4, 5]. Elevated Phe levels exert neurotoxic effects, disrupting brain development and function. Without treatment, PKU can result in severe intellectual disability, epilepsy, behavioral abnormalities, microcephaly, hypopigmentation, and a distinctive musty odor [6, 7].

Early diagnosis via newborn screening is essential to prevent severe neurological manifestations [6, 7]. Despite the introduction of licensed pharmacological therapies, including sapropterin dihydrochloride (BH4), pegvaliase and sepiapterin, dietary management is the foundation therapy for most individuals with PKU, particularly with classical phenotypes who exhibit minimal to no residual PAH activity [8–11]. A rigorous Phe restriction is supplemented with a Phe-free protein substitute and low protein foods [7, 10].

When dietary treatment is initiated early and followed consistently, intellectual development can remain within normal limits. However, dietary adherence declines with increasing age, and subtle neurocognitive, psychological, behavioral and emotional deficits are frequently reported [3, 12–15]. Early reports from the UK and Australia found that only 30% of individuals over 15 years maintained Phe levels within the therapeutic range, compared to 70% in children under 10 years old [16]. These findings have been corroborated by several U.S. and European studies [17–20]. More recently, a large European multicenter study involving 1323 patients, primarily treated with conventional dietary therapy, reported that 73–89% of blood Phe levels were within the recommended range until the end of adolescence [21]. However, this proportion declined to 64% in young adults (19–30 years) and further reduced to 40% in individuals aged 41 years and older. Classical PKU, the most prevalent phenotype (*n* = 625; 48%), presented the greatest challenges in maintaining metabolic control, with only 54% of blood Phe levels within the therapeutic target range [21]. These findings highlight the urgent need for strategies that enhance dietary adherence and metabolic control.

While blood Phe remains the primary biomarker used to monitor metabolic control in PKU, a single measurement, typically obtained after an overnight fast, may not accurately reflect 24-hour metabolic control, as Phe levels can fluctuate significantly throughout the day [22]. In healthy individuals, blood amino acids, including Phe, follow a circadian pattern, generally peaking in the evening [23]. Their fasting Phe levels remain relatively constant, and do not fluctuate by more than 50% over 24-hours [24]. However, patients with PKU, who are consistently adherent to three daily doses of Phe-free protein substitutes, often display an inverse diurnal pattern with higher Phe levels in the early morning, likely due to fasting-induced protein catabolism exceeding protein anabolism overnight [24–27]. This observation is consistent with data showing that prolonged fasting leads to increases in Phe levels, peaking before breakfast [22].

Blood Phe fluctuation (or variability) is important as it may influence intellectual neurocognitive, and psychological outcomes in early and continuously treated individuals with PKU [24–29]. This effect is particularly pronounced in patients with classical PKU [22, 24, 25, 30]. In a study of 16 classical patients with PKU (1–18 years), MacDonald et al. [22] reported a median daily Phe variation of 155 μmol/L, ranging from 80 to 280 μmol/L, a finding that illustrates the challenges of achieving optimal metabolic control even with consistent dietary management. Blood Phe variability is influenced by the dose, timing and frequency of protein substitute intake. Patients with milder PKU and those receiving sapropterin therapy typically have less variability in Phe levels [25, 31–34]. Poor dietary adherence such as inconsistent protein substitute intake or unreported consumption of high Phe foods lead to higher intra-day fluctuations. Furthermore, physiological and dietary factors, including inadequate energy intake, acute illness or inflammatory states, may induce catabolism, resulting in endogenous protein breakdown and elevated plasma Phe levels [24, 35].

Blood Phe stability also reflects broader physiological processes that extend beyond neurocognitive outcomes. Amino acid homeostasis plays a central role in multiple metabolic pathways, including insulin sensitivity [36] and muscle protein synthesis (MPS) [37, 38]. The format in which amino acids are delivered, e.g., intact proteins such as whey versus synthetic free-form amino acid mixtures, can significantly influence MPS by modifying postprandial amino acid availability, a key regulator of anabolic signaling [38]. Conventional amino acid mixtures result in rapid and asynchronous amino acid absorption, leading to variable plasma amino acid concentrations. Such variability can compromise protein synthesis via the “rate-limiting amino acid” phenomenon, in which a deficiency in a single essential amino acid restricts the effective utilization of others [39, 40]. Protein substitute formulations that promote sustained amino acid release and maintain essential amino acid availability may offer physiological benefits. These include more efficient protein utilization, improved nitrogen balance, and enhanced anabolic and enhanced metabolic outcomes in PKU.

Prolonged-release protein substitutes have been investigated as a potential strategy to mitigate diurnal blood Phe variability in individuals with classical PKU. One prolonged release formulation that consists of Phe-free amino acids coated with ethyl cellulose and sodium alginate, produces a fiber-based matrix that enables gradual gastrointestinal release. This controlled absorption replicates the physiological kinetics of intact natural protein sources, promoting more stable plasma amino acid profiles [41–43]. Unlike conventional free L-amino acid-based protein substitutes, which are rapidly absorbed and associated with sharp plasma peaks followed by rapid declines, this protein substitute has a physiological kinetic profile similar to casein, leading to a slower and steady release of amino acids. This more stable absorption profile has the potential to enhance nitrogen retention, increase blood Tyr availability, and reduce oxidative amino acid losses [35, 43, 44].

In PKU, current evidence evaluating prolonged-release protein substitutes remains limited but includes randomized controlled trials, observational studies, and case reports involving both healthy individuals and patients with PKU [43–47]. These have primarily focused on the effects on protein status and nitrogen balance [43] or have assessed product acceptability and gastrointestinal tolerance [45–47]. Despite promising early data, no studies have specifically evaluated if a prolonged-release protein substitute can optimize metabolic control in PKU by minimizing Phe fluctuations following overnight fasting. This period represents the longest catabolic interval within a 24-hour cycle and is associated with elevated endogenous Phe production due to protein breakdown. Furthermore, the potential for broader physiological and clinical benefits, such as more stable blood Tyr levels associated with less Phe variability, remains unexplored.

This study primarily aimed to investigate if administering a prolonged-release protein substitute could improve metabolic control by reducing overnight catabolism and the associated early morning peaks, compared to conventional protein substitutes. Also, its impact was studied on blood Tyr, Phe/Tyr ratio, and branched-chain amino acid (BCAA) levels, which are essential for protein and neurotransmitter synthesis.

## Methods

### Study and control products

The test product, prolonged-release protein substitute (PR-AA; APR Applied Pharma Research, Switzerland), was available in two formats: granules and fruit bars. Both contained the same formulation, consisting of amino acids coated with a fiber-based ethyl cellulose and sodium alginate layer that masks the taste and odor of the free amino acids. This coating technology has been shown to provide a prolonged release of amino acids over a 7-hour period in healthy volunteers [44].

PR-AA granules (15 g protein equivalent per 24 g sachet) were fortified with additional vitamins and minerals while the bars (10 g protein equivalent per 60 g bar or 5 g protein equivalent per 30 g bar) contained amino acids only. The nutritional composition of each formulation is presented in Additional file 1 (Supplementary Table 1).

The study product was administered as a single evening dose during the intervention period. All other daily protein substitute doses were a Phe-free L-amino-acid–based protein substitute (AA) that was taken throughout the study. None of the participants used casein glycomacropeptide (cGMP) as their protein substitute.

### Study participants

Inclusion criteria were children aged 5 to 16 years with a confirmed diagnosis of classical PKU, identified by newborn screening and confirmed with genetic testing [48]. All participants commenced a Phe-restricted diet shortly after birth and were prescribed a Phe-free protein substitute consisting of free amino acids, administered at least three times daily.

Exclusion criteria included a diagnosis of mild phenotype, treatment with sapropterin, neurological problems due to late diagnosis, known hypersensitivity to any excipients or components of the investigational product, pregnancy or breastfeeding during the study period, the presence of a comorbidity (e.g., diabetes, autism), poor adherence with protein substitute use, inability to understand and comply with the study requirements, and participation in any other clinical studies concomitantly or within two weeks prior to study entry.

### Study design

**Fig. 1:**
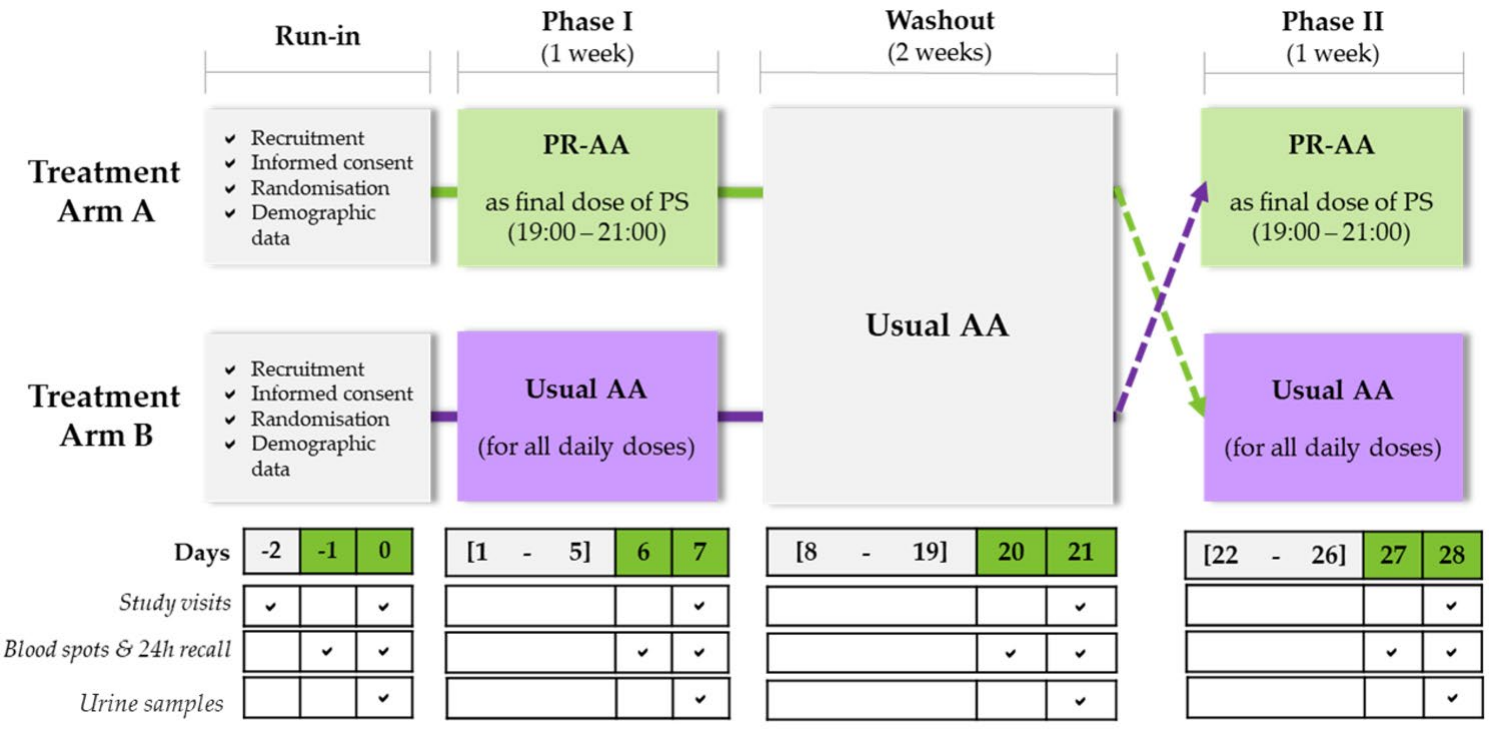
Study design. Abbreviations: PR-AA, prolonged-release amino acid; AA, usual amino acid; PS, protein substitute

This 4-week, randomized, controlled, crossover study was designed to investigate if a PR-AA protein substitute, given as one dose in the evening, could improve metabolic control by reducing overnight catabolism and the associated early morning Phe peaks, compared to conventional protein substitutes (AA) in children with classical PKU (Fig. 1). Patients were randomly assigned to one of two treatment arms in a crossover design:

- *Treatment Arm A:* During the initial 7-day intervention period (days 0 -7) participants received one evening dose of the PR-AA (providing 15-20 g protein equivalent), while continuing their usual protein substitute (AA) for all other daytime doses. The PR-AA dose was equivalent to their usual evening protein substitute dose. After a 2-week washout period (days 8-21), participants crossed over to Treatment Arm B and received their usual AA protein substitute for all daily doses, including the evening dose, for 7 days.
- *Treatment Arm B:* Participants took their usual AA protein substitute for all doses during the 7-day treatment period. Following a 2-week washout period (days 8-21), they changed to Treatment Arm A, and received the PR-AA as their final, evening daily dose for 7 days, following the same protocol described above.

### Randomization and blinding

The patients were randomized to one of the two treatment arms (A or B) which was generated using Statistical Analysis System (SAS). Allocation occurred following confirmation of eligibility and completion of the informed consent process. This was an open-label trial; therefore, blinding of participants, investigators, and outcome assessors was not applicable.

### Interventions

The amount of the study product matched the protein equivalent provided by each participant’s usual AA protein substitute. Patients could choose from two presentations of study product, i.e., granules, or bars.

To ensure accurate metabolic monitoring, the evening dose of the protein substitute (PR-AA or usual AA) was taken between 07:00–09:00 p.m., allowing for an 8–10 hour overnight fast before the collection of morning blood samples via finger-prick at 05:00, 06:00, 07:00 a.m.

### Outcomes

The primary study parameter was the blood Phe level measured before breakfast at 05:00, 06:00, 07:00 a.m., after one-week administration of the PR-AA and AA.

Secondary outcome measures included: blood Tyr, Phe/Tyr ratio, and BCAA levels to evaluate additional metabolic responses to the study product; urinary urea and creatinine levels to assess the study product’s efficacy in reducing nocturnal catabolic episodes; adherence to the prescribed diet; and occurrence of any side effects, particularly gastrointestinal symptoms.

### Data collection

Data was collected between 30 June 2023–24 May 2024. Throughout the trial, participants or their caregivers were informed about the study and trained on specific procedures to ensure consistent data collection and adherence to the study protocol. Participants aged ≥12 years independently completed the study questionnaires. For children younger than 12 years, questionnaires were completed by parents or primary caregivers. In the case of children aged 8–11 years, questionnaires were completed by the child with parental assistance provided as needed.

Each participant was visited 5 times at home: at recruitment, one day before each treatment phase, and at the end of each treatment phase. During the initial visit, eligibility was confirmed, demographic data were collected, and informed consent was obtained. Additionally, participants and caregivers received training on blood spot and urine sample collection and storage at home, as well as detailed guidance on how to accurately complete food diaries and study questionnaires.

During follow-up, trained participants/caregivers collected blood and urine samples, and completed food diaries. All study materials were collected by the investigator after each home visit. All concomitant medications taken during the study were also recorded with indication, dose information, and dates of administration.

#### Blood samples

Participants or caregivers collected three finger-prick morning blood samples on filter cards (Perkin Elmer 226; UK Standard NBS, Public Health England, London, UK) at 05:00, 06:00, 07:00 a.m. prior to breakfast, on two consecutive days before and after each treatment phase. A total of 24 samples were collected:

1. Baseline Days −1, and 0; on usual AA,
2. End of the 1st treatment phase: Days 6 and 7; on usual AA or PR-AA,
3. Baseline of the 2nd treatment phase: Days 20 and 21; on usual AA,
4. End of the 2nd treatment phase: Days 27 and 28; on usual AA or PR-AA.

Samples were returned to the laboratory for analysis of blood Phe, Tyr, and BCAA. Blood Phe and Tyr levels were measured from a 3.2 mm dried blood spot punch using tandem mass spectrometry (MS/MS). Branched-chain amino acids were quantified following phenylisothiocyanate (PITC) derivatization using ultra-performance liquid chromatography (UPLC) and ultraviolet detection.

#### Food diaries

Adherence to the prescribed diet and use of protein substitutes was assessed using a 2-day food diary. Participants/caregivers recorded all food and beverages, including PR-AA and usual AA consumed over two consecutive days before and after each treatment phase (days −1, 0, 6, 7, 20, 21, 27 and 28). Participants were asked to repeat their menus on days −1, 6, 20 and 27 and similarly for days 0, 7, 21 and 28. Only protein exchange foods were calculated.

#### Urine samples

A second void urine sample was done on Days 0, 7, 21, and 28. This sample was frozen at home until collection by the research dietitian.

#### Safety and tolerability assessment

A multi-component approach was used to evaluate the safety and tolerability of the study product. This included adverse event monitoring, gastrointestinal symptom assessment, and protocol adherence. Data on the duration and severity of the adverse events, the need for hospitalization, length of hospital stay, and any treatment-related interruptions were also collected. Palatability was also assessed; however, those results will be reported elsewhere.

### Statistical analysis

#### Sample size calculation

Sample size was determined based on the primary study outcome (change in morning blood Phe variation with study product (PR-AA) in comparison to standard care (usual AA) considering data published by Daly et al. [26] and previous findings related to the study product.

A sample size of 16 patients was determined to be sufficient to demonstrate a significant response of PR-AA versus usual AA, with 80% power and a 5% significance level for a two-sided test, assuming a standard deviation of 40 µmol/L. The sample size calculation was performed using the Proc Power procedure in SAS for a crossover design (paired data).

#### Primary and secondary efficacy analysis

The primary and secondary efficacy analyses were conducted primarily according to the “intention-to-treat” (ITT) principle, followed by a “per-protocol” (PP) analysis to complement the treatment effect estimates obtained from the ITT approach [49, 50].

The primary efficacy endpoint analysis was performed comparing blood Phe levels before and after treatment. This was analyzed using an Analysis of Variance (ANOVA) for a crossover design according to Wallenstein’s method [51], with 95% Confidence Intervals (CIs) for the treatment differences at each time point. Although there was a sufficient washout period between treatments, a pre-test analysis was performed to check for sequence (carry-over) and period effects between the groups before analyzing the treatment effect. In the second stage, the effects of “hour” (time of blood spot collection), “day” (before treatment day vs. end of treatment day), “treatment” (type of product), and their interactions were assessed. The means and the standard errors are derived from ANOVA (Least Square Means).

Among the secondary efficacy endpoints, comparisons were made between treatment groups for blood spot time profiles of amino acids (i.e., inter-day fluctuations) and urine markers (urea and creatinine levels), using ANOVA for a crossover design according to Wallenstein’s method [51]. The 95% Confidence Limits for the differences between treatments were computed.

Additional post-hoc analyses were conducted to investigate trends observed in the primary analysis and graphical representations, as stipulated by the study protocol. These analyses were designed to provide a more in-depth evaluation of specific observations. For some analyses, a posteriori power of the tests was calculated to further validate the findings.

Statistical methods included ANOVA and Analysis of Covariance (ANCOVA) for mixed models, appropriate for the study’s crossover design and repeated measures structure. Student’s t-tests were applied for unpaired comparisons between treatment groups. A specific post-hoc analysis was also performed to assess changes in the Phe/Tyr ratio on days 0, 7, 21, and 28 using ANOVA for repeated measures, accounting for the crossover design. Pearson’s correlation was used to analyze the relationship between blood BCAA level and blood Phe and Tyr levels, as well as between the energy intake from a single dose of PR-AA and blood amino acid levels.

All tests were two-sided, and the level of significance was set at α = 0.05. Data on the outcomes were reported as means (standard deviation), medians (range), 95% confidence intervals, and level of statistical significance. Statistical analyses were performed with SAS, version 9.4, and reported according to the CONSORT (Consolidated Standards of Reporting Trials) statement [52].

### Ethical permission

Ethical approval was given by the North West - Greater Manchester West Research Ethics Committee on 30 April 2022. The Health Research Authority and Health and Care Research Wales Approval Letter was issued on 03 May 2022. Written informed consent was obtained from each participant or by the participant’s legally acceptable representative prior to evaluations performed for eligibility. The study was conducted in accordance with International Conference on Harmonization Good Clinical Practice (ICH GCP E6 R2) and applicable laws and regulations.

## Results

### Participants

Sixteen participants were recruited from a single PKU treatment center (Birmingham Children’s Hospital), as per protocol requirement. Sixteen children (8 males, 8 females) with a mean age of 11.2 ± 3.3 years were selected according to inclusion and exclusion criteria, and were randomized into two treatment sequences. Mean blood Phe levels at diagnosis (1539 ± 454.6 µmol/L; range: 970–2540 µmol/L) and genetic analysis confirmed that all enrolled patients had classical PKU (Additional file 1: Supplementary Table 2).

Three participants were then excluded from the efficacy analysis: two patients due to errors in their blood collection cards and confusion over the time of blood collections, and one failed to take the study product. Thirteen children completed the study in compliance with the protocol (5 males, 8 females), with a mean age of 11.8 ± 3.2 years (range: 7–16 years) (Table 1).

**Table 1:** Subject characteristics, randomization scheme

N	Age (years)	Sex	Phenotype	Ethnicity	Sequence	**1**^**st**^ **Treatment Phase** [Days 1–7]	**2**^**nd**^ **Treatment** Phase [Days 22–28]	Type of study product
1	7	F	Classical	Caucasian	2	AA	PR-AA	Bar
2	7	F	Classical	Caucasian	2	AA	PR-AA	Bar
3	8	F	Classical	Caucasian	1	PR-AA	AA	Bar
4	8	M	Classical	Caucasian	1	PR-AA	AA	Bar
5	10	M	Classical	Caucasian	1	PR-AA	AA	Bar
6	13	F	Classical	Caucasian	1	PR-AA	AA	Bar
7	13	M	Classical	Caucasian	1	PR-AA	AA	Bar
8	14	F	Classical	British Pakistani	2	AA	PR-AA	Granules
9	14	F	Classical	British Pakistani	2	AA	PR-AA	Granules
10	14	F	Classical	Caucasian	1	PR-AA	AA	Bar
11	15	M	Classical	Caucasian	1	PR-AA	AA	Bar
12	15	M	Classical	Caucasian	2	AA	PR-AA	Bar
13	16	F	Classical	Caucasian	2	AA	PR-AA	Bar

Abbreviations: M, male; F, female; PR-AA, prolonged-release amino acid; AA, usual amino acid

The majority of the children were of Caucasian origin (*n=* 11, 85%), and two were British-Pakistani. Two patients received granules as the study product, and eleven patients took bars. Protein equivalent and tyrosine intakes from both study and control products are provided in Additional file 1 (Supplementary Table 3).

At baseline, one patient reported issues with constipation, and another a recent history of tonsillitis.

### Primary efficacy endpoint: blood phenylalanine profile

**Fig. 2:**
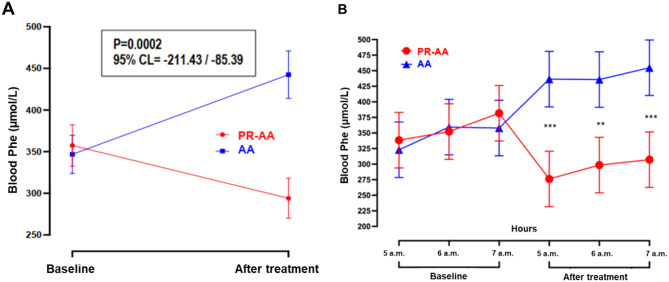
Blood Phe profile (mean ± SE) at baseline and at the end of 1-wk treatment with PR-AA vs. AA stratified by study day (**A**), and by morning hours and study day (**B**). ANOVA for mixed models; ** corresponds to *p* < 0.02, and *** corresponds to *p* < 0.01. Abbreviations: PR-AA, prolonged-release amino acid; AA, usual amino acid; Phe, phenylalanine; CL, confidence level; SE, standard error

Table 2 and Fig. 2A summarize the blood Phe levels measured early in the morning at baseline and after one week of treatment with the PR-AA and AA. Treatment with PR-AA led to a statistically significant reduction in morning blood Phe levels (mean difference: 63.6 ± 28.93 µmol/L; −17.8%; *p =* 0.0484), whereas treatment with AA led to a significant increase (mean difference from baseline: 95.5 ± 28.9 µmol/L; +27.6%; *p =* 0.0063). At the end of treatment, comparison between groups confirmed statistically significant lower morning blood Phe levels for the PR-AA (mean difference: 148.41 ± 28.93 μmol/L; *p =* 0.0002).

**Table 2:** Blood Phe levels (µmol/L) measured early in the morning at baseline and after one week of treatment with the PR-AA vs. AA

Treatment period:	Blood Phe, μmol/L				Mean difference (± SE) between groups at the end of treatment
	**Mean** ± **SD**		Median [range]		
	PR-AA (N = 13)	AA (N = 13)	PR-AA (N = 13)	AA (N = 13)	
Baseline	357.5 ± 155.6	346.8 ± 143.7	415.0 [68 - 725]	357.0 [62 - 602]	148.41 ± 28.93
End of treatment	294.0 ± 149.9	442.4 ± 177.6	299.0 [40 - 517]	428.0 [167 - 930]	
*P-value*	**0.0484**	**0.0063**			**0.0002**

ANOVA for mixed models. Abbreviations: PR-AA, prolonged-release amino acid; AA, usual amino acid; Phe, phenylalanine; N, number; SD, standard deviation; SE, standard error.

The variation in morning blood Phe levels measured at 05:00, 06:00, 07:00 a.m. was also examined both at baseline and after one week of treatment (Fig. 2B, Table 3). Although minor differences across the three morning time points were noted within each treatment group, they were not pronounced.

**Table 3:** Blood Phe levels at baseline vs. after one week of treatment with the PR-AA vs. AA, stratified by blood spot collection time (05:00, 06:00, 07:00 a.m.)

Treatment	Period	Hours	N of blood spots	Blood Phe level (μmol/L)	
				Mean ± SD	Median [range]
**PR-AA**	Baseline	5 a.m.	13	338.4 ± 145.33	418 [68–496]
		6 a.m.	13	352.3 ± 146.49	429 [86–515]
		7 a.m.	13	381.9 ± 181.65	415 [96–725]
	End of treatment	5 a.m.	13	276.2 ± 149.37	299 [40–491]
		6 a.m.	13	298.5 ± 155.94	298 [65–517]
		7 a.m.	13	307.2 ± 154.91	312 [71–501]
**AA**	Baseline	5 a.m.	13	323.1 ± 141.86	348 [62–558]
		6 a.m.	13	359.5 ± 145.17	410 [78–574]
		7 a.m.	13	357.9 ± 152.60	359 [87–602]
	End of treatment	5 a.m.	13	436.5 ± 193.54	434 [167–930]
		6 a.m.	13	435.8 ± 178.13	413 [197–858]
		7 a.m.	13	454.8 ± 174.43	428 [215–852]

Abbreviations: PR-AA, prolonged-release amino acid; AA, usual amino acid; N, number; SD, standard deviation; Phe, phenylalanine

**Fig. 3:**
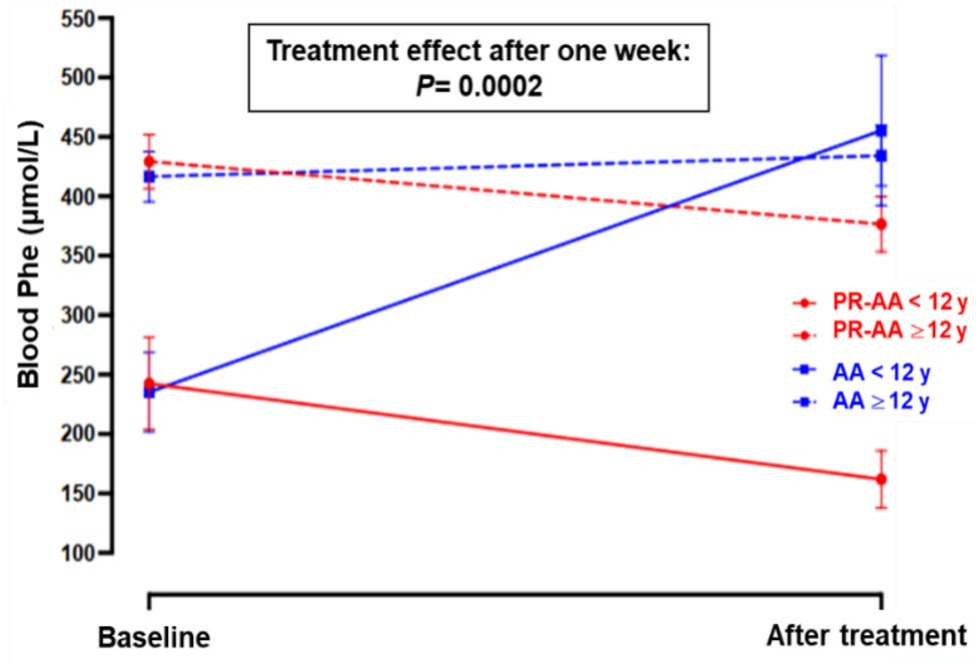
Blood Phe profile (mean ± SE) at baseline and at the end of 1-wk treatment with PR-AA vs. AA stratified by age. ANCOVA for mixed models, using age as a covariate. Abbreviations: PR-AA, prolonged-release amino acid; AA, usual amino acid; Phe, phenylalanine; SE, standard error

To assess the impact of age on treatment response, patients were stratified into two age groups: <12 years (*n=* 5) and ≥12 years (*n=* 8) (Fig. 3, Table 4). At baseline, children in the younger age group (<12 years) had significantly lower morning blood Phe levels compared to children ≥12 years in both treatment arms (*p* < 0.0001). After treatment, PR-AA resulted in a decrease in mean blood Phe levels in both age groups; however, the magnitude of change was more noticeable in younger children (mean difference: 80.6 μmol/L; 33.2%) compared to those ≥12 years (mean difference: 52.9 μmol/L; 12.3%). Conversely, AA resulted in an increase in blood Phe levels in both age groups. Even after adjusting for age, using a PR-AA product sustained lower early morning Phe levels compared to AA (*p =* 0.0002).

**Table 4:** Blood Phe levels at baseline and after one week of treatment with PR-AA vs. AA across different age groups (<12 years vs. ≥12 years)

Treatment period:	Blood Phe, μmol/L								**Age-adjusted mean difference (± SE) between groups at the end of treatment**^**b**^
	**Mean** ± **SD**				Median [range]				
	PR-AA		AA		PR-AA		AA		
	< 12 years (N = 5)	≥ 12 years (N = 8)	< 12 years (N = 5)	≥ 12 years (N = 8)	< 12 years (N = 5)	≥ 12 years (N = 8)	< 12 years (N = 5)	≥ 12 years (N = 8)	
Baseline	242.5 ± 149.9	429.4 ± 110.9	235.3 ± 129.3	416.6 ± 103.7	211 [68–442]	448 [245–725]	189 [62–417]	399 [252–602]	148.41 ± 28.9
End of treatment	161.9 ± 94.2	376.5 ± 115.1	455.5 ± 244.6	434.2 ± 124.5	143 [40–292]	392 [127–517]	408 [167–930]	446 [256–621]	*p*= **0.0002**
Mean difference (± SE) between age groups in each treatment group ^a^:									
Baseline	186.9 ± 41.8		181.3 ± 37.5						
P-value	**<0.0001**		**<0.0001**						
End of treatment	214.7 ± 35.4		21.3 ± 59.1						
P-value	**<0.0001**		0.7571						

^a^Student *t-*test for unpaired data

^b^ANCOVA for mixed models, with age as a covariate

Abbreviations: PR-AA, prolonged-release amino acid; AA, usual amino acid; Phe, phenylalanine; N, sample size; SD, standard deviation; SE, standard error

A subgroup analysis was performed to assess whether treatment efficacy was influenced by the severity of baseline (pre-treatment) Phe elevation. A threshold of 360 µmol/L (>360 µmol/L vs. ≤360 µmol/L) was used, as this is a widely accepted clinical cut-off in the management of PKU. After adjusting for baseline Phe, the difference between the two treatment groups at the end of the study period remained statistically significant (*p* = 0.0163) (Additional file 1: Supplementary Table 4 and Supplementary Figure 1). A second model examined the treatment effect exclusively in patients with baseline Phe level of >360 μmol/L. Although no significant difference was observed at baseline (*p* = 0.1743), PR-AA resulted in a significantly greater reduction in blood Phe levels at the end of treatment compared to the AA (mean difference: 110.12 ± 41.87 μmol/L; *p* = 0.0339).

### Secondary efficacy endpoints

#### Blood tyrosine profile

**Fig. 4:**
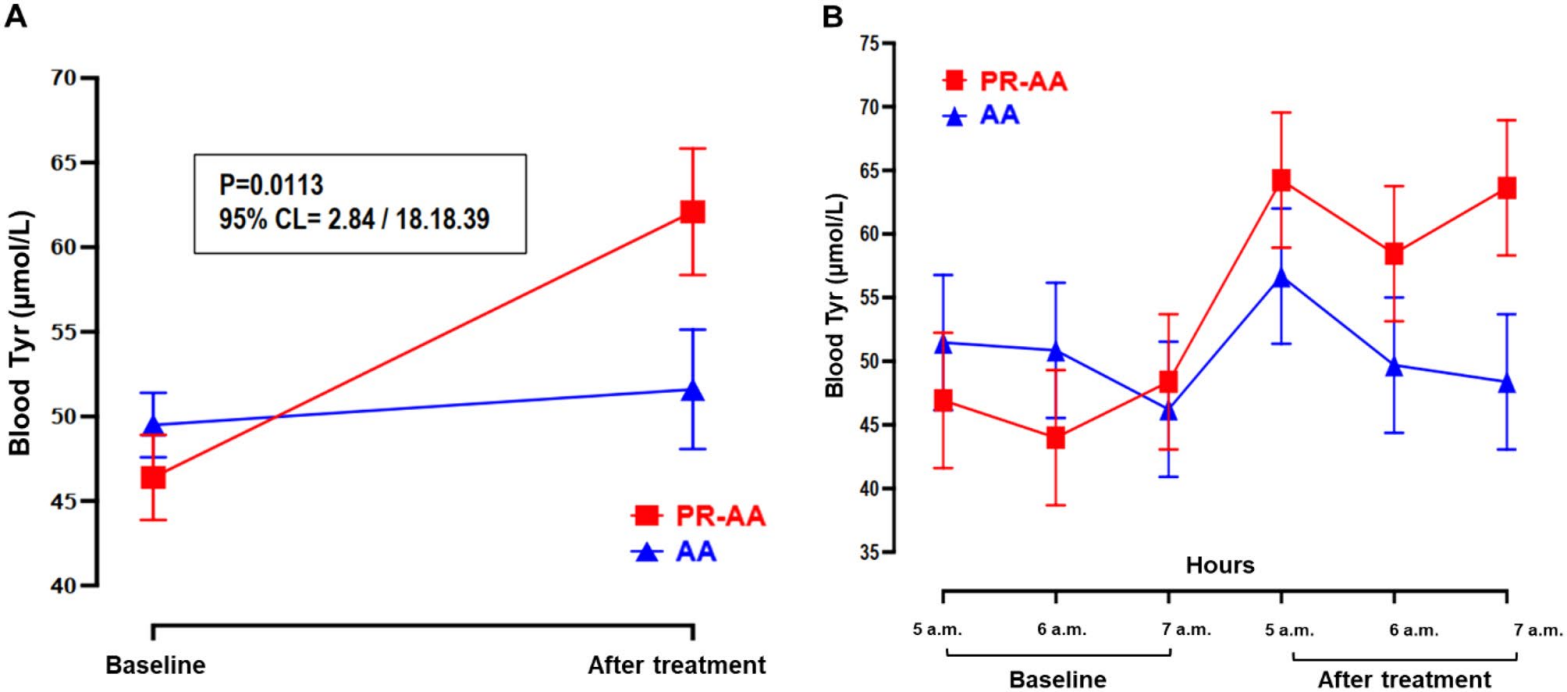
Blood Tyr profile (mean ± SE) at baseline and at the end of 1-wk treatment with PR-AA vs. AA stratified by study day (**A**), and by morning hours and study day (**B**). ANOVA for mixed models. Abbreviations: PR-AA, prolonged-release amino acid; AA, usual amino acid; Tyr, tyrosine; CL, confidence level; SE, standard error

Figure 4A and Table 5 present the results for blood Tyr levels measured early in the morning at baseline and after one week of treatment with PR-AA and AA. The only significant increase over time was observed for the PR-AA treatment (mean difference: 15.67 ± 3.52 µmol/L; +33.8%; *p =* 0.0008), whereas the AA treatment showed no significant change (mean difference: 2.08 ± 3.52 µmol/L; +4.2%; *p* = 0.5660). At the end of one-week treatment period, PR-AA showed significantly higher morning blood Tyr levels compared to the AA (62.1 µmol/L vs. 51.6 µmol/L; mean difference: 10.51 ± 3.52; *p =* 0.0113).

**Table 5:** Blood Tyr levels (µmol/L) measured early in the morning at baseline and after one week of treatment with the PR-AA (study product) vs. AA

Treatment period:	Blood Tyr, μmol/L				Mean difference (± SE) between groups at the end of treatment
	**Mean** ± **SD**		Median [range]		
	PR-AA (N = 13)	AA (N = 13)	PR-AA (N = 13)	AA (N = 13)	
Baseline	46.44 ± 15.76	49.55 ± 11.93	45 [24–97]	51 [30–88]	10.51 ± 3.52
End of treatment	62.10 ± 23.43	51.66 ± 22.02	55 [38–159]	44 [27–133]	
*P-value*	**0.0008**	0.5660			**0.0113**

ANOVA for mixed models. Abbreviations: PR-AA, prolonged-release amino acid; AA, usual amino acid; Tyr, tyrosine; N, number; SD, standard deviation; SE, standard error

No significant difference was observed in Tyr levels among the three morning time points in both treatment arms (*p =* 0.3962) (Fig. 4B and Table 6).

**Table 6:** Blood Tyr levels at baseline vs. after one week of treatment with the PR-AA vs. AA, stratified by blood spot collection time (05:00, 06:00, 07:00 a.m.)

Treatment	Period	Hours	N of blood spots	Blood Tyr level (μmol/L)	
				Mean ± SD	Median [range]
PR-AA	Baseline	5 a.m.	13	46.9 ± 5.3	48 [24–71]
		6 a.m.	13	44.0 ± 10.6	45 [27–59]
		7 a.m.	13	48.4 ± 22.1	45 [25–97]
	End of treatment	5 a.m.	13	64.2 ± 19.7	59 [41–110]
		6 a.m.	13	58.5 ± 17.2	54 [38–92]
		7 a.m.	13	63.6 ± 32.1	55 [38–159]
AA	Baseline	5 a.m.	13	51.5 ± 14.6	51 [32–88]
		6 a.m.	13	50.8 ± 10.6	53 [30–70]
		7 a.m.	13	46.2 ± 10.4	41 [32–62]
	End of treatment	5 a.m.	13	56.7 ± 27.2	47 [34–133]
		6 a.m.	13	49.7 ± 22.4	45 [27–109]
		7 a.m.	13	48.4 ± 16.0	43 [31–81]

Abbreviations: PR-AA, prolonged-release amino acid; AA, usual amino acid; N, number; SD, standard deviation; Tyr, tyrosine

**Fig. 5:**
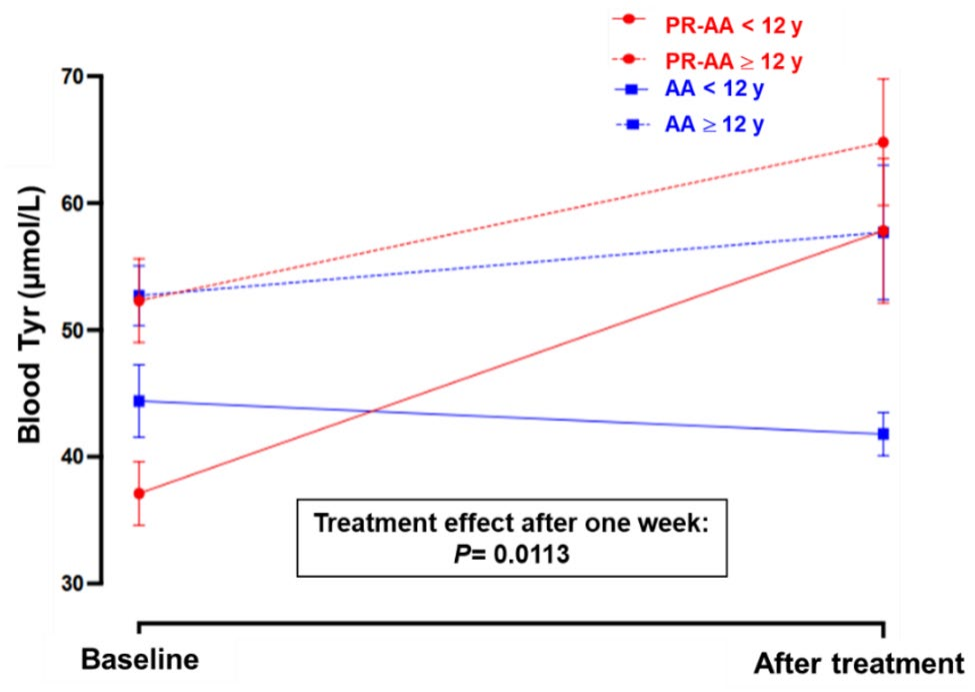
Blood Tyr profile (mean ± SE) at baseline and at the end of 1-wk treatment with PR-AA vs. AA stratified by age. ANCOVA for mixed models, using age as a covariate. Abbreviations: PR-AA, prolonged-release amino acid; AA, usual amino acid; Tyr, tyrosine; SE, standard error

Stratifying the sample based on age (<12 years vs. ≥12 years) revealed that baseline mean morning Tyr levels were significantly higher in older children aged ≥12 years compared to the younger ones with both the PR-AA (*p =* 0.008) and AA (*p =* 0.0324). After using the PR-AA for one week, morning blood Tyr levels increased in both age groups, with a greater increase in younger patients (mean difference: 20.7 μmol/L; 55.8%) compared to older children (mean difference: 12.5 μmol/L; 23.9%) (Fig. 5, Table 7). However, as older children had higher baseline levels and showed a relatively smaller increase compared to younger children, mean morning Tyr levels became comparable between the two age groups after one week of treatment with PR-AA (57.8 ± 22.03 µmol/L vs. 64.8 ± 24.33 µmol/L; *p =* 0.3717).

**Table 7:** Blood Tyr levels at baseline and after one week of treatment with PR-AA vs. AA across different age groups (<12 years vs. ≥12 years)

Treatment period:	Blood Tyr, μmol/L								**Age -adjusted mean difference (± SE) between groups at the end of treatment**^**b**^
	**Mean** ± **SD**				Median [range]				
	PR-AA		AA		PR-AA		AA		
	< 12 years (N = 5)	≥ 12 years (N = 8)	< 12 years (N = 5)	≥ 12 years (N = 8)	< 12 years (N = 5)	≥ 12 years (N = 8)	< 12 years (N = 5)	≥ 12 years (N = 8)	
Baseline	37.1 ± 9.75	52.3 ± 16.14	44.4 ± 11.09	52.7 ± 11.52	34.0 [24–58]	48.5 [31–97]	40.0 [30–64]	52.0 [36–88]	10.51 ± 3.52
End of treatment	57.8 ± 22.03	64.8 ± 24.33	41.8 ± 6.56	57.7 ± 25.95	48.0 [38–110]	57.5 [41–159]	42.0 [32–56]	48.0 [27–133]	*p*= **0.0113**
Mean difference (±SE) between age groups in each treatment group ^a^:									
Baseline	15.12 ± 4.63		8.31 ± 3.74						
P-value	**0.008**		**0.0324**						
End of treatment	6.99 ± 7.73		15.91 ± 6.86						
P-value	0.3717		**0.008**						

^a^Student *t-*test for unpaired data

^b^ANCOVA for mixed models, with age as a covariate

Abbreviations: Tyr, tyrosine; N, sample size; SD, standard deviation; PR-AA, prolonged-release amino acid; AA, usual amino acid; SE, standard error

Treatment with AA led to a slight decrease in mean morning Tyr levels in younger children (mean difference: 2.6 μmol/L; −5.9%), and a modest increase in older children (mean difference: 5 μmol/L; +9.5%). At the end of treatment, the difference in morning Tyr levels between two age groups remained significant for AA, with higher levels observed in older (≥12 years) compared to younger children (*p =* 0.0080).

Overall, these findings suggest that PR-AA provided a more consistent and pronounced increase in morning blood Tyr levels regardless of age, whereas the AA resulted in smaller and more variable changes, particularly showing a greater response in older patients.

A subgroup analysis of Tyr levels, stratified by baseline Phe using a 360 µmol/L threshold as a covariate, indicated that baseline Phe levels did not significantly affect the Tyr profile (*p =* 0.2294), suggesting that the observed differences in morning Tyr levels with PR-AA and AA treatments occurred independently of baseline blood Phe levels (Additional file 1: Supplementary Table 5).

#### Phenylalanine/Tyrosine (Phe/Tyr) ratio

**Fig. 6:**
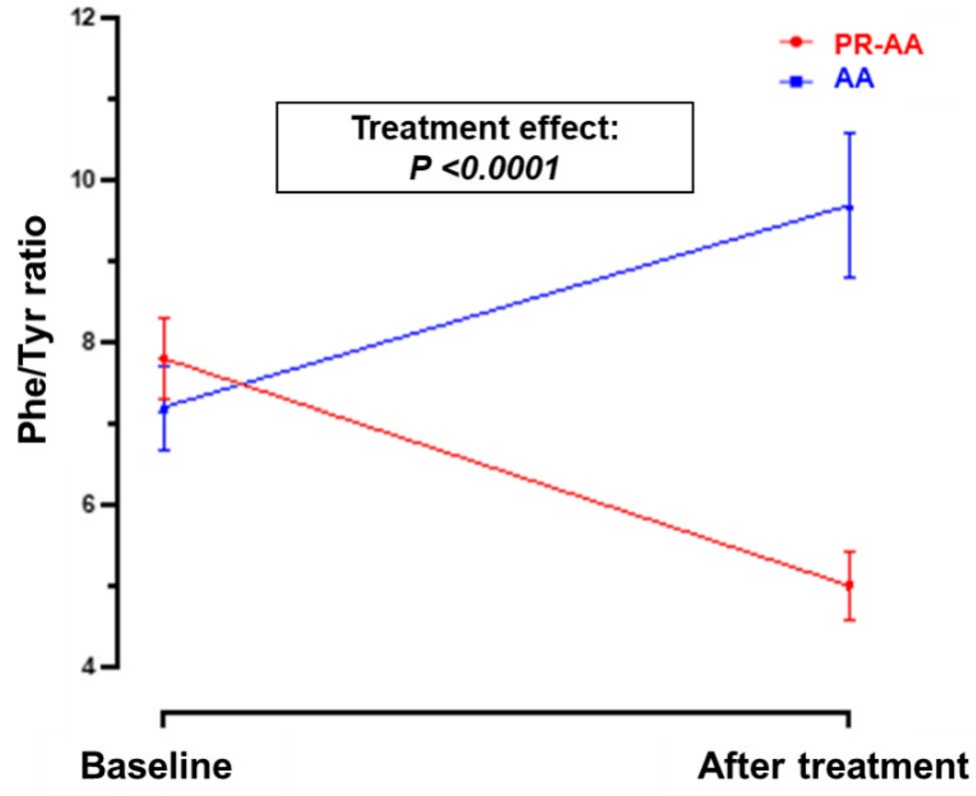
Phe/Tyr ratio (mean ± SE) at baseline and at the end of 1-wk treatment with PR-AA vs. AA. ANOVA for mixed models. Abbreviations: PR-AA, prolonged-release amino acid; AA, usual amino acid; SE, standard error; Phe, phenylalanine; Tyr, tyrosine

The Phe/Tyr ratio was assessed at baseline and after one week of treatment with either PR-AA or AA (Table 8, Fig. 6). Baseline values were similar in both groups. At the end of one week, the change in Phe/Tyr ratio differed significantly between the two treatments (*p =* 0.0005). Within group analysis confirmed that the use of a PR-AA resulted in a statistically significant decrease (mean difference: 2.8; −36%; *p =* 0.0042), compared to an increase in the AA treatment mean difference: 2.5; +35%; *p =* 0.0084).

**Table 8:** Phe/Tyr ratio at baseline and after one week of treatment with PR-AA vs. AA

Treatment period:	*Phe/Tyr*				Mean difference between groups at the end of treatment
	**Mean** ± **SD**		Median [range]		
	PR-AA (N = 13)	AA (N = 13)	PR-AA (N = 13)	AA (N = 13)	
Baseline	7.8 ± 3.11	7.2 ± 3.23	8.0 [1.9–13.8]	7.7 [1.6–13.9]	4.7
End of treatment	5.0 ± 2.59	9.7 ± 5.57	5.1 [0.4–9.8]	8.2 [3.2–26.8]	
*P-value*	**0.0042**	**0.0084**			**<0.0001**

ANOVA for mixed models. Abbreviations: PR-AA, prolonged-release amino acid; AA, usual amino acid; Phe, phenylalanine; Tyr, tyrosine; N, number; SD, standard deviation; SE, standard error

#### Blood profile of branched-chain amino acids (BCAA)

Branched-chain amino acids (BCAAs), leucine, isoleucine, and valine, are involved in protein synthesis, energy production, and metabolic regulation. In this study, BCAA profiles were assessed as a secondary endpoint. Table 9 summarizes morning blood BCAA levels at baseline and at the end of treatment for the two treatment groups. Additionally, the correlations between blood BCAA levels and Phe and Tyr levels were explored across both treatments and are presented in Additional file 1 (Supplementary Table 6).

**Table 9:** Blood leucine, valine and isoleucine levels (µmol/L) and total BCAA measured early in the morning at baseline and after one week of treatment with PR-AA vs. AA

	Blood Leucine (μmol/L)		Blood Valine (μmol/L)		Blood Isoleucine (μmol/L)		Blood BCAA (μmol/L)	
	PR-AA (N = 13)	AA (N = 13)	PR-AA (N = 13)	AA (N = 13)	PR-AA (N = 13)	AA (N = 13)	PR-AA (N = 13)	AA (N = 13)
**Mean ± SD blood level, µmol/L**								
Baseline	107.0 ± 21.8	103.3 ± 21.9	224.3 ± 47.6	214.7 ± 52.6	59.7 ± 17.5	60.4 ± 17.0	391.1 ± 79.9	378.4 ± 85.1
End of treatment	112.4 ± 34.5	101.7 ± 26.4	193.7 ± 56.9	216.3 ± 62.1	64.4 ± 25.8	57.1 ± 15.2	370.5 ± 113.6	375.1 ± 98.1
* P-value*	0.2044	0.7187	**0.0009**	0.8232	0.1343	0.2938	0.1704	0.8053
**Median [range] of blood level**								
Baseline	109 [74–177]	99 [72–151]	221 [146–351]	200 [136–338]	59 [34–121]	58 [39–91]	378 [266–649]	360 [256–562]
End of treatment	106 [72–241]	93 [62–150]	183 [120–345]	202 [119–344]	55 [32–174]	53 [31–88]	338 [241–760]	370 [212–574]
**Between-treatment comparison of BCAA levels, mean ± SE:**								
End of treatment, µmol/L	10.86 ± 4.2		21.97 ± 6.89		7.64 ± 3.08		3.45 ± 13.32	
* P-value*	**0.0238**		**0.0078**		**0.0290**		0.7996	

ANOVA for mixed models. Abbreviations: PR-AA, prolonged-release amino acid; AA, usual amino acid; BCAA, branched-chain amino acids; N, number; SD, standard deviation; SE, standard error

A statistically significant decrease in valine levels was observed (although valine remained within clinical reference ranges) with PR-AA treatment (*p =* 0.0009); however, there was no significant change in other BCAA levels from baseline to the end of treatment period with both PR-AA and AA. Between-group comparisons did not identify clinically relevant differences in total BCAA levels (Table 9). Furthermore, no correlation was observed between morning blood BCAA and Phe levels, and no strong overall correlation was found between BCAA and Tyr levels (Additional file 1: Supplementary 6). These findings suggest that single dose administration of the PR-AA formulation does not substantially alter systemic BCAA levels or their relationship with Phe and Tyr.

#### Urinary urea and creatinine

Table 10 presents data on urinary urea and creatinine levels measured at baseline and after one week of treatment with PR-AA and AA. Due to the difficulty in collecting samples, data were available for a limited number of patients. While slight changes were observed over time in both treatment groups, the differences between them were not pronounced. Using the PR-AA substitute, mean urinary urea and creatinine levels remained relatively stable, whereas a minor decrease was noted with the AA treatment. Considerable individual variability was observed in both groups at both time points.

**Table 10:** Urine urea and creatinine concentrations at baseline and after one week of treatment with PR-AA vs. AA

Treatment	Urine testing day	Urine urea (μmol/L)				Urine creatinine (μmol/L)	
		N	Mean (SD)	Median [range]	N	Mean (SD)	Median [range]
**PR-AA**	Baseline	10	292.6 ± 170.2	285 [70–562]	11	7.4 ± 3.8	7.0 [2.5–14.3]
	End of treatment	12	287.8 ± 129.1	306 [59–524]	10	7.8 ± 5.7	7.8 [1.2–21.5]
**AA**	Baseline	13	316.2 ± 141.5	346 [54–525]	12	10.8 ± 6.0	9.1 [1.8–24.4]
	End of treatment	12	273.7 ± 167.2	223 [55–588]	10	6.7 ± 3.8	6.6 [2.3–13.9]

Abbreviations: PR-AA, prolonged-release amino acid; AA, usual amino acid; N; number of patients; SD, standard deviation

#### Energy intake from protein substitutes

Mean energy intake from the PR-AA (293 kcal/day) was more than twice that of AA (119 kcal/day) (Table 11). Despite this difference, no significant effects were observed on blood Phe (*p =* 0.7468) or Tyr levels (*p =* 0.4397). The absence of a correlation between energy intake from the PR-AA and blood amino acid levels supports the ANCOVA results, indicating that energy intake from a single evening dose of PR-AA did not significantly influence blood Phe or Tyr levels (data not shown). It is important to note that only protein exchange foods were measured, and although dietary assessments were consistent (Additional file 1: Supplementary Table 7), the overall influence of energy intake on blood amino acid levels could not be clearly determined.

**Table 11:** Energy intake (kcal/day) from PR-AA vs. AA

Treatment	N	Mean (SD)	Median [range]
**PR-AA**	13	292.6 ± 96.52	350 [89.1–350.0]
**AA**	13	119.4 ± 75.89	100 [10.0–350.0]

Abbreviations: PR-AA, prolonged-release amino acid; AA, usual amino acid; N; number of patients; SD, standard deviation

#### Safety and tolerability

Minor adverse events were reported during the intervention phase in five patients (Additional file 1: Supplementary Table 8). These were all mild and likely associated with common viral infections (e.g., sore throat, gastroenteritis), consistent with the study being conducted during the winter season. Gastrointestinal symptoms, such as bloating, distension, burping, flatulence, regurgitation, and abdominal discomfort or pain, remained unchanged from baseline (Additional file 1: Supplementary Table 9). These symptoms were not attributed to the study product and were considered endemic within the school environment.

#### Adherence

All 13 patients included in the analysis adhered to the prescribed intake of the study product. Two additional subjects, excluded from the analysis due to blood spot collection errors, also followed the dosing regimen as instructed. One participant was classified as non-adherent, having declined to take the study product.

#### Concomitant medication

During the study period, five children received concomitant medications. One patient was prescribed two laxatives, while another received three medications, including intravenous glucose with saline, an antiemetic, and an analgesic/antipyretic (the latter had a viral gastroenteritis [negative for *Clostridium difficile*] during the washout period requiring intravenous fluids and antiemetic treatment; diarrhea resolved within 48 hours). Among the remaining three children, two were prescribed with an analgesic/antipyretic, and one received an antipyretic.

## Discussion

The main objective of PKU treatment is to prevent adverse neurocognitive and psychological outcomes by maintaining blood Phe levels within the therapeutic targets of 120–360 μmol/L in children up to 12 years and 120–600 μmol/L in adolescents [7]. More recently, blood Phe instability and variability have been associated with executive dysfunction, mood disturbances, and reduced treatment efficacy, and Phe fluctuations across the life span have been shown to affect cognitive performance more than Phe levels per se [53, 54]. These findings led the latest European PKU guideline to highlight the need to define recommended targets for Phe fluctuations [7]. However, maintaining stable blood Phe levels over a 24-hour period remains a significant challenge in the management of PKU, particularly in individuals with classical PKU, who exhibit heightened sensitivity to dietary change and protein catabolism. These patients often experience marked intra- and inter-day fluctuations in blood Phe levels, despite adherence to prescribed dietary protocols [21, 22, 25, 30]. Considering the potential detrimental effects of cumulative day-to-day Phe fluctuations on cognitive performance over months and years, there is an apparent need for practical, evidence-based strategies to minimize Phe fluctuations, without increasing patient or caregiver burden.

This randomized, controlled crossover trial is the first to specifically evaluate the benefits of a prolonged-release protein substitute for lowering or stabilizing early morning blood Phe levels in patients with classical PKU, that may offer an adjunctive therapeutic benefit by stabilizing blood Phe levels while maintaining blood Phe levels within the recommended target range. The intervention was compared against conventional protein substitutes composed of free amino acids. The study demonstrated that a single evening dose of the PR-AA led to an approximately 18% statistically significant reduction in early morning blood Phe levels, resulting in a mean Phe level of 294 µmol/L. In contrast, the conventional protein substitute resulted in elevated Phe levels, with a mean of 442 µmol/L, exceeding the recommended target range of 120–360 µmol/L. The findings from this short-term study highlight the limitations of standard, rapidly absorbed protein substitutes in maintaining overnight blood Phe control, but support that PR-AA may help achieve metabolic stability during the overnight fasting period, a time that is usually associated with Phe accumulation in classical PKU.

Subgroup analyses indicated that patients with elevated baseline Phe levels (over the upper target level of > 360 µmol/L) experienced a more substantial reduction when treated with PR-AA, whereas in those receiving the conventional AA, blood Phe levels increased. Younger patients (<12 years) responded more favorably compared to adolescents, exhibiting greater reductions in mean blood Phe levels with the PR-AA. Median blood Phe levels also showed a consistent decrease with the PR-AA in both age groups, accompanied by a narrower range, indicating reduced variability. In contrast, both mean and median blood Phe levels increased with the conventional AA, particularly in children < 12 years (from a mean and median level of 235 and 189 µmol/L to 456 and 408 µmol/L), and the range widened substantially (from 62 to 417 µmol/L to 167–930 µmol/L; Table 4), suggesting blood Phe instability and greater interindividual variability. While elevated early morning Phe levels observed with conventional AA may reflect differences in pharmacokinetic profiles, other factors may also have contributed to metabolic variability, including interindividual variation in amino acid absorption and metabolism [55, 56], inconsistencies in dietary adherence, timing and frequency of amino acid intake [22, 25, 31] or variable intake of energy or natural protein/Phe sources [57]. Overall, given the small sample size, these subgroup findings should be interpreted with caution, as limited participant numbers reduce statistical power and increase susceptibility to individual variability. Although these exploratory observations provide preliminary insights, these should be confirmed in larger, adequately powered studies.

In addition to lowering morning blood Phe, PR-AA consistently increased blood Tyr levels across all age groups. This contrasted with the variable or minimal Tyr responses observed with the conventional protein substitute. The dual effect of reduced Phe and increased Tyr resulted in a clinically significant improvement in the Phe/Tyr ratio, potentially an important biomarker for metabolic control. It is possible that an improved Phe/Tyr ratio may support dopaminergic and noradrenergic function, particularly in classical PKU [58–60].

Considering the challenges with maintaining optimal metabolic control and the associated neurocognitive outcomes in PKU, the findings of this study may have meaningful implications for clinical management and therapeutic strategies. During extended fasting periods, such as overnight, endogenous protein catabolism contributes to elevated circulating Phe levels, disproportionately affecting individuals with classical PKU [22, 27, 61]. Uneven intake or poor adherence to protein substitutes during the day can further amplify fluctuations. The latter phenomenon is largely attributed to the absorption kinetics of conventional free amino acid mixtures, that differ significantly from those of intact proteins [39]. Free amino acids are absorbed rapidly, causing early plasma peaks followed by steep declines. This non-physiological absorption pattern has been linked to metabolic imbalance and increased nitrogen loss, particularly when amino acid availability exceeds the capacity for protein synthesis [40, 62].

Another consequence of elevated or fluctuating Phe levels is the impaired transport of other large neutral amino acids (LNAAs), such as Tyr and tryptophan, across the blood brain barrier via competitive inhibition at the L-type amino acid transporter 1 (LAT1) [63, 64]. As precursors for dopamine, serotonin, and norepinephrine, reduced cerebral availability of these LNAAs may compromise monoaminergic neurotransmitter synthesis [64–69]. These findings are supported by studies demonstrating that elevated Phe, high Phe/Tyr ratio and Phe fluctuations are associated with adverse neurocognitive and psychological outcomes [30, 53, 54, 59, 60, 70–74]. Studies in children and adolescents with PKU suggested that greater Phe variability and elevated Phe/Tyr ratio may be linked to lower IQ scores [30, 53, 73, 75] or poor executive function [59, 60, 70]. However, findings were inconsistent, as some reported no association [76] and others showed weak or non-significant correlations [77, 78].

Blood Phe variability and Phe/Tyr ratio have also been linked to anxiety and depression symptoms in adolescents with PKU [71, 79]. Furthermore, there is evidence to show that blood Phe variability is a better predictor of cognitive and executive performance, particularly during later developmental periods [53]. Several studies by Romani et al. [54, 74, 80] demonstrated that fluctuations during childhood or adolescence can predict adult neuropsychological performance and language outcomes. A Phe standard deviation (SD) of <180 μmol/L has been proposed to optimize executive function [54]. More recently, greater Phe variability has been associated with poorer performance in certain neurocognitive domains and possibly with the severity of brain structural damage in adults with PKU [81].

Strategies that reduce Phe variability and improve the Phe/Tyr ratio are increasingly recognized as critical for preserving long-term neurocognitive function. In this study, despite being administered once daily and over a short period, the PR-AA led to favorable shifts in blood Phe, Tyr, and the Phe/Tyr ratio, suggesting a potential to aid long-term neurocognitive outcome. Current European PKU guidelines recommend dividing Phe-free or low-Phe protein substitutes into 3 or more daily doses to reduce 24-hour Phe fluctuations [7]. However, frequent dosing regimens are associated with declining adherence, particularly in adolescents and adults [82–84]. Sensory aversions, including unpleasant taste and odor of conventional protein substitutes further compromise long-term adherence [85]. In PKU, prolonged-release protein substitutes are a useful addition to treatment approaches by helping improve metabolic control but without increasing the daily amount or number of doses of protein substitute required. Their neutral taste and smell may support better adherence.

This study evaluated blood BCAA levels, and urinary urea and creatinine excretion to explore if the PR-AA offered additional benefits for protein metabolism, nitrogen retention and muscle protein synthesis compared to conventional protein substitutes. Only minor changes in individual BCAA levels were observed in both treatment groups, with no significant differences in total BCAA levels. Urinary urea and creatinine excretion patterns were comparable between groups, indicating no notable differences in nitrogen balance or renal handling. Under the conditions studied, PR-AA therapy did not confer measurable advantages in BCAA availability or nitrogen excretion relative to standard formulations. While the intervention demonstrated benefits in blood Phe control and Phe/Tyr ratio, its impact on anabolic efficiency and nitrogen utilization remains inconclusive. Further investigation using longer-term protocols, is warranted to clarify potential effects on protein accretion and metabolic efficiency.

Energy intake is an important factor in contributing to Phe stability. However, this study only evaluated energy intake from protein substitutes, and failed to show any associations with energy intake from protein substitutes and blood amino acids. More studies are needed to review the impact of energy intake on Phe stability.

The prolonged-release AA demonstrated a favorable safety and tolerability profile, with no serious adverse events attributed to the intervention. Mild gastrointestinal symptoms were slightly more frequent in the PR-AA treatment but they were considered unrelated to the intervention. Short term treatment adherence was high, and the bar formulation received positive feedback for taste and texture, important factors in long-term adherence in clinical practice.

This study has several limitations that should be considered when interpreting the findings. The study was conducted in a small sample, which is a common constraint in research on rare metabolic disorders. Intensive sampling schedule, particularly the requirement for early morning blood collection (5 a.m.), created further feasibility constraints to achieve a larger sample size. The short intervention duration and single-dose design limit the assessment of long-term efficacy, safety, and sustainability of metabolic improvements. The subgroup analyses, while informative, may be underpowered to detect subtle differences across age groups or baseline Phe levels. Although favorable effects on blood Phe and Tyr levels were observed, the absence of neurocognitive assessments and objective biomarkers of brain health precludes conclusions about functional benefit. Energy intake was not comprehensively measured, and although diets were consistent during the study period, with the exception of the protein substitutes under study, differences in energy contribution from the substitutes may have influenced metabolic outcomes, highlighting the need for more controlled studies. Lack of pharmacokinetic profiling limits mechanistic insight into absorption, distribution, and metabolic action. It is important to note that this study solely focused on the short-term Phe variability and the acute impact of a prolonged-release protein substitute on daily Phe fluctuations, rather than evaluating the long-term effects on steady-state Phe kinetics which may require a substantially longer intervention and wash-out period [86], particularly in individuals with classical PKU.

However, several methodological strengths help mitigate these limitations regarding the small sample size and generalizability of the results. The study utilized a within-subject (repeated-measures) design, which enhances statistical power in small cohorts by minimizing inter-individual variability, as each participant serves as their own control. All enrolled participants had classical PKU, resulting in a highly homogeneous study population and strengthening internal validity. Regular home visits supported adherence and ensured consistent, high-quality sample collection. Collectively, these methodological strengths increase the reliability of the observed physiological responses and provide valuable effect-size estimates to guide the design of future larger, multicenter studies.

Further evaluation in longer-term, real-world studies is warranted to assess sustained acceptability and patient preferences across diverse age groups, and to explore its use during pregnancy, with exercise, and in periods of illness.

## Conclusions

This short-term randomized, controlled crossover study conducted in a single metabolic centre has demonstrated that a once-daily, evening dose of a prolonged-release protein substitute significantly stabilizes early morning blood Phe levels in children with classical PKU. Compared to conventional protein substitutes consisting of free amino acids, the PR-AA formulation was associated with: lower morning Phe levels and variability during extended overnight fasting, increased Tyr levels and improved Phe/Tyr ratio, a key marker of metabolic control. These outcomes suggest that the prolonged-release protein substitute may help nocturnal management of blood Phe control. Further studies with larger cohorts, longer follow-up, and inclusion of neurodevelopmental assessments are warranted to validate these findings across different age groups, optimize the formulation, and confirm sustained metabolic and neurocognitive benefits in the long-term. Additionally, future research should also evaluate nutritional adequacy such as micronutrient status, overall amino acid profiles, growth or body composition as well as adherence to chronic and full-dose administration of PR-AA products.

## Electronic supplementary material

Below is the link to the electronic supplementary material.

Supplementary Material 1

## Data Availability

The data used and/or analyzed in the current study are available from the corresponding author on reasonable request.

## Abbreviations

PKU: Phenylketonuria
Phe: Phenylalanine
PR-AA: Prolonged-release amino acid
AA: Amino acid
Tyr: Tyrosine
BCAA: Branched-chain amino acid
PAH: Phenylalanine hydroxylase
BH4: Sapropterin dihydrochloride
MPS: Muscle protein synthesis
PS: Protein substitute
QRE: Questionnaire
MS/MS: Tandem mass spectrometry
PITC: Phenylisothiocyanate (PITC)
UPLC: Ultra-performance liquid chromatography
ITT: Intention-to-treat
PP: Per protocol
CI: Confidence Interval
ANOVA: Analysis of Variance
ANCOVA: Analysis of Covariance
CONSORT: Consolidated Standards of Reporting Trials
SD: Standard deviation
SE: Standard error
LNAAs: Large neutral amino acids
LAT1: L-type amino acid transporter
