# Correction: Randomized investigation to evaluate phenylalanine fluctuation after overnight fasting in PKU patients treated with prolonged-release versus standard amino acid protein substitute

**DOI:** 10.1186/s13023-026-04386-3

**Published:** 2026-07-31

**Authors:** Anne Daly, Fatma Ilgaz, Sharon Evans, Catherine Ashmore, Alex Pinto, Anita MacDonald

**Affiliations:** 1 Birmingham Children’s Hospital, Birmingham, B4 6NH UK; 2 Department of Nutrition and Dietetics, Faculty of Health Sciences, Hacettepe University, Ankara, 06100 Turkey

**Correction: Orphanet Journal of Rare Diseases (2026) 21:127**

10.1186/s13023-026-04264-y

Following publication of the original article [1], we have been notified that Table 8 heading was published incorrectly.

It is now:

Blood Tyr

It should be:

Phe/Tyr

