# Supplementary material for: Randomized investigation to evaluate phenylalanine fluctuation after overnight fasting in PKU patients treated with prolonged-release versus standard amino acid protein substitute
Source: Orphanet J Rare Dis. 2026 Apr 2;21:127. doi: 10.1186/s13023-026-04264-y (PMC13047778; doi:10.1186/s13023-026-04264-y)
Supplement: Supplementary file 1 — Supplementary Material 1 [file 13023_2026_4264_MOESM1_ESM.docx]

**SUPPLEMENTARY INFORMATION**

**Additional file 1.**

**Supplementary Table 1.** Nutrition declaration of test products.

| **Component, units** | **PKU GOLIKE**  **3-16 granules** | | **PKU GOLIKE BAR**  **(red fruits 60 g)** | | **PKU GOLIKE BAR (tropical 30 g)** | |
| --- | --- | --- | --- | --- | --- | --- |
|  | **Per 100 g** | **Per sachet of 24 g** | **Per 100 g** | **Per bar of 60 g** | **Per 100 g** | **Per bar of 30 g** |
| **Energy and macronutrient** |  |  |  |  |  |  |
| Energy, kcal (kJ) | 280 (1187) | 67 (286) | 292 (1236) | 175 (742) | 275 (1170) | 83 (351) |
| Fat, grams | 0 | 0 | 0 | 0 | 0 | 0 |
| *of which saturates, g* | 0 | 0 | 0 | 0 | 0 | 0 |
| Carbohydrate, g | 4.3 | 1.0 | 53 | 32 | 50 | 15 |
| *of which sugars, g* | 0 | 0 | 34 | 20 | 34 | 10 |
| Fiber, g | 7.1 | 1.7 | 5.4 | 3.3 | 3.6 | 1.1 |
| Protein equivalent^[[1]](#footnote-1)^, g | 62.2 | 15 | 17 | 10 | 17 | 5 |
| Salt, g | 0.06 | 0.015 | 0.08 | 0.05 | 0.30 | 0.09 |
| **Amino acids** |  |  |  |  |  |  |
| L-serine, g | 2.5 | 0.6 | 0.7 | 0.4 | 0.7 | 0.2 |
| L-threonine, g | 3.8 | 0.9 | 1.1 | 0.6 | 1.1 | 0.3 |
| L-leucine, g | 8.6 | 2.1 | 2.4 | 1.4 | 2.4 | 0.7 |
| Glycine, g | 3.8 | 0.9 | 1.1 | 0.6 | 1.1 | 0.3 |
| L-alanine, g | 2.3 | 0.5 | 0.6 | 0.4 | 0.6 | 0.2 |
| L-arginine, g | 3.0 | 0.7 | 0.8 | 0.5 | 0.8 | 0.3 |
| L-cystine, g | 1.5 | 0.4 | 0.4 | 0.3 | 0.4 | 0.1 |
| L-glutamine, g | 15.0 | 3.6 | 4.2 | 2.5 | 4.2 | 1.2 |
| L-histidine, g | 2.1 | 0.5 | 0.6 | 0.4 | 0.6 | 0.2 |
| L-aspartic acid, g | 4.5 | 1.1 | 1.2 | 0.7 | 1.2 | 0.4 |
| L-proline, g | 4.5 | 1.1 | 1.2 | 0.7 | 1.2 | 0.4 |
| L-isoleucine, g | 4.1 | 1.0 | 1.1 | 0.7 | 1.1 | 0.3 |
| L-lysine, g | 5.3 | 1.3 | 1.5 | 0.9 | 1.5 | 0.4 |
| L-tryptophan, g | 1.5 | 0.4 | 0.4 | 0.3 | 0.4 | 0.1 |
| L-valine, g | 3.8 | 0.9 | 1.1 | 0.6 | 1.1 | 0.3 |
| L-methionine, g | 1.0 | 0.3 | 0.3 | 0.2 | 0.3 | 0.1 |
| L-tyrosine, g | 7.5 | 1.8 | 2.1 | 1.2 | 2.1 | 0.6 |
| L-phenylalanine, mg |  |  | 25 | 15 | 25 | 8 |
| **Vitamins** |  |  |  |  |  |  |
| Vitamin A (RE), µg | 1295 | 311 | - | - | - | - |
| Vitamin D, µg | 25 | 6.0 | - | - | - | - |
| Vitamin E, mg | 13 | 3.2 | - | - | - | - |
| Vitamin K, µg | 100 | 24 | - | - | - | - |
| Vitamin C, mg | 135 | 32.31 | - | - | - | - |
| Thiamin, mg | 2.0 | 0.5 | - | - | - | - |
| Riboflavin, mg | 1.9 | 0.5 | - | - | - | - |
| Niacin, mg | 27 | 6.4 | - | - | - | - |
| Vitamin B6, mg | 2.6 | 0.6 | - | - | - | - |

**Supplementary Table 1.** Cont’.

| **Component, units** | **PKU GOLIKE**  **3-16 granules** | | **PKU GOLIKE BAR**  **(red fruits 60 g)** | | **PKU GOLIKE BAR (tropical 30 g)** | |
| --- | --- | --- | --- | --- | --- | --- |
|  | **Per 100 g** | **Per sachet of 24 g** | **Per 100 g** | **Per bar of 60 g** | **Per 100 g** | **Per bar of 30 g** |
| Folic acid, µg | 267 | 64.1 | - | - | - | - |
| Vitamin B12, µg | 4.2 | 1.0 | - | - | - | - |
| Biotin, µg | 54 | 13 | - | - | - | - |
| Pantothenic acid, mg | 11 | 2.6 | - | - | - | - |
| **Minerals** |  |  |  |  |  |  |
| Potassium, mg | 1250 | 300 | - | - | - | - |
| Calcium, mg | 1339 | 321 | - | - | - | - |
| Magnesium, mg | 304 | 72.9 | - | - | - | - |
| Phosphorus, mg | 1060 | 254 | - | - | - | - |
| Chloride, mg | 0.75 | 0.18 | - | - | - | - |
| Sodium, mg | 25 | 5.9 | - | - | - | - |
| Iron, mg | 23 | 5.6 | - | - | - | - |
| Zinc, mg | 14 | 3.4 | - | - | - | - |
| Copper, mg | 1.4 | 0.3 | - | - | - | - |
| Manganese, mg | 2.5 | 0.6 | - | - | - | - |
| Selenium, µg | 58 | 14 | - | - | - | - |
| Chromium, µg | 46 | 11 | - | - | - | - |
| Molybdenum, µg | 88 | 21 | - | - | - | - |
| Iodine, µg | 225 | 54.0 | - | - | - | - |
| **Others** |  |  |  |  |  |  |
| Carnitine, g | 0.08 | 0.02 | 0.02 | 0.014 | 0.02 | 0.007 |
| Taurine, g | 0.21 | 0.05 | 0.06 | 0.035 | 0.06 | 0.018 |
| Choline, g | 321 | 77.1 | - | - | - | - |
| Inositol, g | 214 | 51.4 | - | - | - | - |

**Supplementary Table 2.** Demographic characteristics and genetic mutations of study participants.

| **N** | **Age (years)** | **Sex** | **Phenotype** | **Ethnicity** | **Mutations** | |
| --- | --- | --- | --- | --- | --- | --- |
|  |  |  |  |  | **Allele 1** | **Allele 2** |
| 1 | 7 | F | Classical | Caucasian | c.?-510_706-?del | c.1222C>T p.(Arg408Trp) |
| 2 | 7 | F | Classical | Caucasian | c.754C>T p.(Arg252Trp) | c.1315+1G>A p.? |
| 3 | 8 | F | Classical | Caucasian | c.1066-11G>A p.? | c.1222C>T p.(Arg408Trp) |
| 4 | 8 | M | Classical | Caucasian | c. 1315+1 G>A p. ? | c. 1315+1 G>A p. ? |
| 5 | 10 | M | Classical | Caucasian | c.1066-11G>A p.? | c.1222C>T p.(Arg408Trp) |
| 6 | 13 | F | Classical | Caucasian | c.1222C>T p.(Arg408Trp) | c.782G>A p.(Arg261Gln) |
| 7 | 13 | M | Classical | Caucasian | c.1222 C>T p.Arg408Trp | c.1222 C>T p.Arg408Trp |
| 8 | 14 | F | Classical | British Pakistani | c.558_559del p.(Trp187Glyfs*12) | c.558_559del p.(Trp187Glyfs*12) |
| 9 | 14 | F | Classical | British Pakistani | c.558_559del (Trp187Glyfs*12) | c.558_559del p.(Trp187Glyfs*12) |
| 10 | 14 | F | Classical | Caucasian | Not done^a^ | Not done^a^ |
| 11 | 15 | M | Classical | Caucasian | c. 1066-11 G>A p. ? | c. 912+1 G>A p. ? |
| 12 | 15 | M | Classical | Caucasian | c.1222C>T p.(Arg408Trp) | c.1315+1G>A p.? |
| 13 | 16 | F | Classical | Caucasian | c. 1066-11G>A | c. 1066-11G>A |

^a^ One female subject declined confirmatory molecular testing; however, her clinical characteristics strongly support classical PKU.

**Supplementary Table 3.** Protein equivalent and Tyr intake from study and control products.

| **N** | **1^st^ Treatment**  **Phase**  **[Days 1-7]** | **2^nd^ Treatment**  **Phase**  **[Days 22-28]** | **Type of study product** | **Tyr intake (g/day)** | | | **Protein equivalent intake from PR-AA (g/day)** |
| --- | --- | --- | --- | --- | --- | --- | --- |
|  |  |  |  | **Baseline**  **with AA** | **During study with AA** | **During study**  **with PR-AA** |  |
| 1 | AA | PR-AA | Bar | 5.6 | 3.8 | 2.4 | 15 |
| 2 | AA | PR-AA | Bar | 8.5 | 6.4 | 2.4 | 20 |
| 3 | PR-AA | AA | Bar | 11.3 | 8.5 | 2.4 | 20 |
| 4 | PR-AA | AA | Bar | 5.6 | 3.8 | 2.4 | 20 |
| 5 | PR-AA | AA | Bar | 11.3 | 8.5 | 2.4 | 20 |
| 6 | PR-AA | AA | Bar | 8.5 | 5.7 | 2.4 | 20 |
| 7 | PR-AA | AA | Bar | 11.3 | 8.5 | 2.4 | 20 |
| 8 | AA | PR-AA | Granules | 8.5 | 5.7 | 2.4 | 20 |
| 9 | AA | PR-AA | Granules | 8.5 | 5.7 | 2.4 | 20 |
| 10 | PR-AA | AA | Bar | 10.0 | 6.7 | 1.8 | 20 |
| 11 | PR-AA | AA | Bar | 8.2 | 6.2 | 2.4 | 20 |
| 12 | AA | PR-AA | Bar | 11.3 | 8.5 | 2.4 | 20 |
| 13 | AA | PR-AA | Bar | 5.6 | 3.8 | 2.4 | 20 |

Abbreviations: Tyr, tyrosine; PR-AA, prolonged-release amino acid; AA, usual amino acid.

**Supplementary Table 4.** Blood Phe levels at baseline and after one week of treatment with PR-AA and AA across two baseline Phe levels (≤ 360 μmol/L vs. > 360 μmol/L).

| **Treatment period:** | **Blood Phe, μmol/L** | | | | | | | | **Phe-adjusted mean difference (± SE) between groups at the end of treatment ^a^** |
| --- | --- | --- | --- | --- | --- | --- | --- | --- | --- |
|  | **Mean** ± **SD** | | | | **Median [range]** | | | |  |
|  | **PR-AA** | | **AA** | | **PR-AA** | | **AA** | |  |
|  | **≤ 360 μmol/L**  **(N=5)** | **> 360 μmol/L**  **(N=8)** | **≤ 360 μmol/L**  **(N=5)** | **> 360 μmol/L**  **(N=8)** | **≤ 360**  **μmol/L**  **(N=5)** | **> 360**  **μmol/L**  **(N=8)** | **≤ 360 μmol/L**  **(N=5)** | **> 360**  **μmol/L**  **(N=8)** |  |
| Baseline | 196.4±109.5 | 451.1±68.2 | 285.2±143.4 | 387.8±134.2 | 268 [74-288] | 434 [362-558] | 333 [96-452] | 495 [306-586] | 148.41 ± 53.14  ***P=* 0.0163** |
| End of treatment | 179.7±169.4 | 365.4±90.9 | 389.3±277.7 | 475.5±90.9 | 140 [59-475] | 349 [244-491] | 286 [193-880] | 395 [134-563] |  |

^a^ ANCOVA for mixed models, with baseline Phe levels as a covariate.

Abbreviations: PR-AA, prolonged-release amino acid; AA, usual amino acid; Phe, phenylalanine; N, sample size; SD, standard deviation; SE, standard error.

**
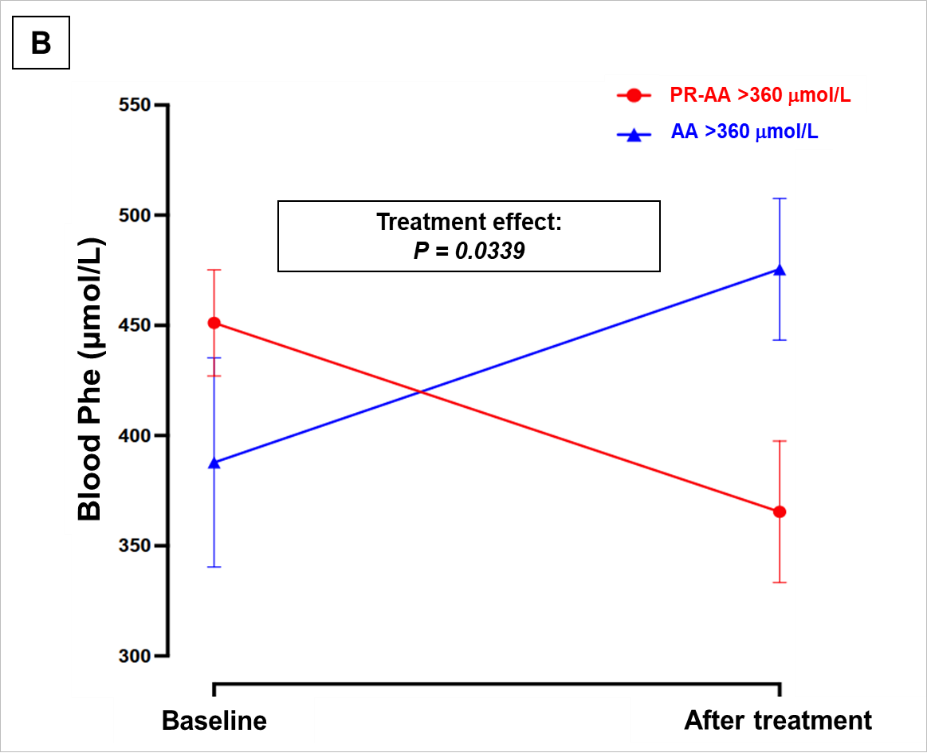

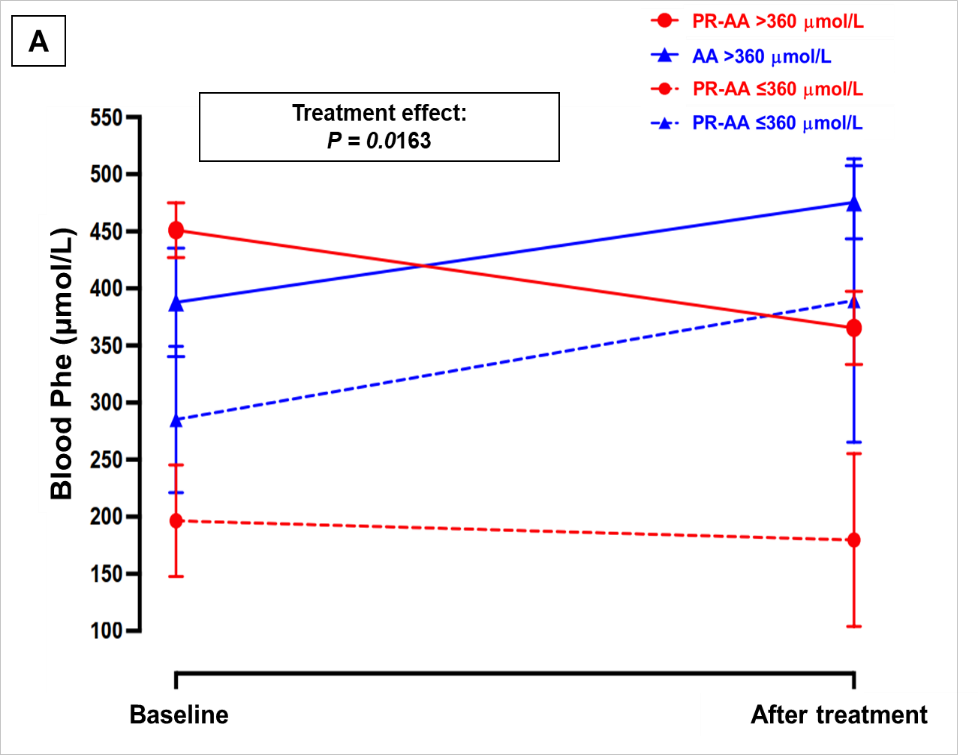
**

**Supplementary Figure 1.** Blood Phe profile stratified by baseline Phe level (>360 μmol/L vs. ≤360 μmol/L), ANCOVA for mixed models, with baseline blood Phe level as a covariate (**A**); blood Phe profile of patients with baseline Phe level of >360 μmol/L, ANOVA for mixed models (**B**). Abbreviations: Phe, phenylalanine; PR-AA, prolonged-release amino acid; AA: usual amino acid.

**Supplementary Table 5.** Blood Tyr levels at baseline and after one week of treatment with PR-AA vs. AA across two baseline Phe levels (≤ 360 μmol/L vs. > 360 μmol/L).

| **Treatment period:** | **Blood Tyr, μmol/L** | | | | | | | | **Phe-adjusted mean difference (± SE) between groups at the end of treatment ^a^** |
| --- | --- | --- | --- | --- | --- | --- | --- | --- | --- |
|  | **Mean** ± **SD** | | | | **Median [range]** | | | |  |
|  | **PR-AA** | | **AA** | | **PR-AA** | | **AA** | |  |
|  | **≤ 360 μmol/L**  **(N=5)** | **> 360 μmol/L**  **(N=8)** | **≤ 360 μmol/L**  **(N=5)** | **> 360 μmol/L**  **(N=8)** | **≤ 360**  **μmol/L**  **(N=5)** | **> 360**  **μmol/L**  **(N=8)** | **≤ 360 μmol/L**  **(N=5)** | **> 360**  **μmol/L**  **(N=8)** |  |
| Baseline | 41.88±8.53 | 54.22±18.4 | 45.22±10.74 | 49.33±4.82 | 43.6 [28 – 52] | 50.3 [31 – 93] | 41.6 [34 – 62] | 48.8 [43 – 59] | -10.51±5.42  *P=* 0.0764 |
| End of treatment | 56.11±21.07 | 65.99±15.84 | 48.44±17.67 | 53.66±23.47 | 49.0 [40 – 93] | 66.3 [41 – 89] | 45.3 [35 – 79] | 46.3 [31 – 107] |  |

^a^ ANCOVA for mixed models, with baseline Phe levels as a covariate.

Abbreviations: PR-AA, prolonged-release amino acid; AA, usual amino acid; Tyr, tyrosine; Phe, phenylalanine; N, sample size; SD, standard deviation; SE, standard error.

**Supplementary Table 6.** Correlation of blood BCAA levels with blood Phe and Tyr, stratified by treatment and study time point.

|  | **Treatment** | **Blood testing day** | **Blood BCAA** | | |
| --- | --- | --- | --- | --- | --- |
|  |  |  | ***r*** | ***P*** | **95% CI** |
| Blood Phe | PR-AA | Baseline | 0.3376 | **0.0356** | [0.0247, 0.5902] |
|  |  | At the end of treatment | 0.5456 | **0.0004** | [0.0069, 0.5780] |
|  | AA | Baseline | 0.3210 | **0.0463** | [0.2736, 0.7368] |
|  |  | At the end of treatment | -0.0072 | 0.9653 | [-0.3220, 0.3090] |
| Blood Tyr | PR-AA | Baseline | 0.2893 | 0.0740 | [-0.0289, 0.5543] |
|  |  | At the end of treatment | 0.1870 | 0.2608 | [-0.1411, 0.4781] |
|  | AA | Baseline | 0.2274 | 0.1639 | [-0.0949, 0.5065] |
|  |  | At the end of treatment | 0.3482 | **0.0298** | [0.0368, 0.5980] |

Correlations calculated using Pearson’s r. Statistically significant values are shown in bold. Abbreviations: PR-AA, prolonged-release amino acid; AA, usual amino acid; BCAA, brached-chain amino acids; Phe, phenylalanine; Tyr, tyrosine; CI, confidence interval.

**Supplementary Table 7.** Mean energy intakes at the end of each treatment phase (on test days) and mean difference in energy intakes between the two study periods.

| **Subjects** | **Test Days** | | **Mean difference**  **(kcal)** | **Mean difference**  **(%)** |
| --- | --- | --- | --- | --- |
|  | **Assessment 1 (Kcal/day)** | **Assessment 2 (Kcal/day)** |  |  |
| **1** | 1775 | 1892 | -117 | 7 |
| **2** | 1531 | 1620 | -89 | 6 |
| **3** | 1463 | 1485 | -22 | 2 |
| **4** | 1741 | 1724 | 17 | 1 |
| **5** | 1754 | 1891 | -137 | 8 |
| **6** | 1013 | 1087 | -74 | 7 |
| **7** | 1627 | 1456 | 171 | 10 |
| **8** | 1247 | 1260 | -13 | 1 |
| **9** | 1340 | 1249 | 91 | 7 |
| **10** | 1165 | 1146 | 19 | 2 |
| **11** | 1201 | 1237 | -36 | 3 |
| **12** | 1328 | 1256 | 72 | 5 |
| **13** | 1584 | 1424 | 160 | 10 |
| **Mean ± SD** | 1444 ± 248 | 1441 ± 271 |  | 5% ± 3% |

**Supplementary Table 8.** Summary of adverse events (AEs) recorded during the study.

| **N** | **Treatment at time of recording** | **Description of AE and symptoms** | **Severity of AE** | **Presence of serious AE** | **Relationship with treatment** | **Action taken** | **Date of onset and resolution** | **Date of hospitalization and discharge** | **Outcome** |
| --- | --- | --- | --- | --- | --- | --- | --- | --- | --- |
| N=1 | AA | Viral infection  Symptoms: Sore throat and high fever | Mild | No | Not Related | Interrupted temporarily until recovery | 08/02/2024 – 15/02/2024 | - | Resolved |
| N=1 | PR-AA | Viral illness  Symptoms: Vomiting and high fever | Mild | No | Not Related | Interrupted temporarily,  resumed on 19/02/2024, with blood samples collected on 24/02/2024 and 25/02/2024. | 08/02/2024 – 10/02/2024 | - | Resolved |
| N=1 | PR-AA | Viral illness  Symptoms: Vomiting and high fever | Mild | No | Not Related | Interrupted temporarily,  resumed on 19/02/2024, with blood samples collected on 24/02/2024 and 25/02/2024. | 08/02/2024 – 10/02/2024 | - | Resolved |
| N=1 | PR-AA | Illness  Symptoms: Diarrhea and vomiting | Mild | Yes* | Not Related | Interrupted temporarily, hospitalization required with IV fluids; resumed after one week. | 16/03/2024 – 23/03/2024 | 17/03/2024 - 23/03/2024 | Resolved |
| N=1 | PR-AA | Illness  Symptoms: Vomiting | Mild | No | Not Related | Discontinued permanently due to an inability to tolerate the study product | 29/05/2024 – 29/05/2024 | - | Resolved |

Abbreviations: PR-AA, prolonged-release amino acid; AA, usual amino acid; N, number of patients; AE, adverse events.

One participant experienced a sore throat during the washout period and therefore delayed the study for one week. Phe levels during illness were 570, 800, 540, and 380 µmol/L. The study resumed once levels had returned to the acceptable range (after reaching 380 µmol/L). One participant was admitted to hospital with gastroenteritis prior to commencing the second phase of the study. Phe levels during illness were 750 and 600 µmol/L, which subsequently decreased to 100 µmol/L before restarting the study. The study was also delayed for two additional participants because their mother was unwell (the children themselves were not ill).

**Supplementary Table 9.** Gastrointestinal tolerance parameters during the study periods.

|  | **N (%) of subjects affected** | **Treatment at the time of**  **adverse events** | | **N (%) of subjects with adverse events due to treatment** | |
| --- | --- | --- | --- | --- | --- |
|  |  | **PR-AA** | **AA** | **PR-AA** | **AA** |
| Diarrhea and/or constipation | N=1 (6.3%) | 1 | - | - | - |
| Bloating and/or distension | - | - | - | - | - |
| Nausea and/or vomiting | N=4 (25.0%) | 4 | - | - | - |
| Burping/ flatulence/ regurgitation | - | - | - | - | - |
| Abdominal discomfort/pain | - | - | - | - | - |

Abbreviations: PR-AA, prolonged-release amino acid; AA, usual amino acid; N, number of patients; AE, adverse events.

1. 1g of protein equivalent = 1.2 g of AAs.

   The protein content is provided by the amino-acids. [↑](#footnote-ref-1)
